# Impact of variations in ALD procedure on nanomorphology, protecting properties and chemical stability of thin TiO_2_ films

**DOI:** 10.1039/d5ra09703g

**Published:** 2026-02-12

**Authors:** Hana Krýsová, Tomáš Imrich, Hana Tarábková, Pavel Janda, Josef Krýsa

**Affiliations:** a J. Heyrovský Institute of Physical Chemistry of the Czech Academy of Sciences Dolejškova 2155/3 182 23 Prague 8 Czech Republic hana.tarabkova@jh-inst.cas.cz; b Department of Inorganic Technology, University of Chemistry and Technology Technická 5 166 28 Prague 6 Czech Republic josef.krysa@vscht.cz

## Abstract

Thin TiO_2_ films were deposited by atomic layer deposition (ALD) at 150 and 250 °C on FTO and Si/SiO_2_ substrates to examine the effect of deposition conditions on morphology, structure, chemical stability, and photoelectrochemical performance. Films grown at 150 °C were amorphous and crystallised into anatase after annealing at 500 °C, accompanied by nanoscale morphological rearrangement. In contrast, films deposited at 250 °C were amorphous and non-stoichiometric (TiO_2−*x*_) with Ti^3+^ self-doping; annealing reduced the doping level without inducing crystallisation. The films degraded in 0.1 M HClO_4_ within 72 h but remained stable in alkaline media (pH 8). Electrochemical studies using the [Fe(CN)_6_]^3−/4−^ redox couple showed that low-temperature ALD TiO_2_ layers (8–50 nm) effectively blocked charge transfer, whereas this approach was unsuitable for high-temperature ALD films due to self-doping. The as-deposited high-temperature ALD TiO_2_/FTO exhibits higher photoelectrochemical (PEC) efficiency than low-temperature films due to Ti^3+^ self-doping. The as-deposited low-temperature ALD TiO_2_/FTO shows negligible PEC efficiency, which increases significantly after annealing owing to the formation of the anatase phase.

## Abstract 2

Thin TiO_2_ films were deposited by atomic layer deposition (ALD) at 150 and 250 °C on FTO and Si/SiO_2_ substrates to examine the effect of deposition conditions on morphology, structure, chemical stability, and photoelectrochemical performance.

## Graphical abstract

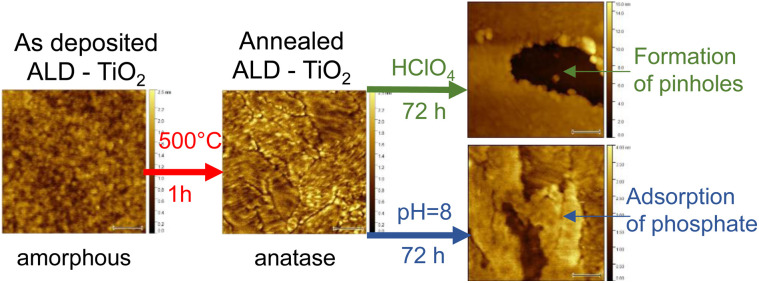

## Introduction

Surface coverage by thin films for the improvement of mechanical, optical, and electrochemical properties of solid surfaces is of great technological importance. In this context, corrosion, which is an electrochemical process, represents one of the concerns. If not properly protected, in an oxidizing and humid atmosphere, disintegration of metallic structural elements into oxides takes place. Protection of semiconductors against corrosion and photocorrosion in liquid media represents another important research topic.^1–4^ In photoelectrochemical applications of semiconductors, light-generated charge carriers can lead to decomposition of the material. One way of protecting a semiconductor in contact with a liquid is to cap it with a thin film, such that front side illumination is not attenuated. Insulating or wide-bandgap semiconducting oxides such as titanium dioxide are preferred as capping layer materials. Regarding a suitable deposition technique, atomic layer deposition (ALD) is the only method that enables homogeneous, pinhole free, continuous, and conformal coating of complex, three-dimensional substrates.^5^

Recently, titanium dioxide overlayer prepared by the ALD coating technique (deposition temperature 150 °C) has been investigated with the aim to protect a semiconducting hematite electrode against corrosion and photocorrosion.^6–8^ Depending on the thickness of the protecting TiO_2_ layer, the passage of electrical current was progressively hindered as the layer thickness was increased from 2 to 8 nm. Such behavior was explained by the non-favourable valence band positions of hematite and titania.^6^ Moehl *et al.* investigated ALD TiO_2_ protective overlayers for light absorbers in photoelectrochemical water-splitting devices, where fluorine-doped SnO_2_ (FTO) was used as a model substrate.^9^ ALD deposition was performed at temperatures 120 and 150 °C, such overlayers need at least 40–60 nm thickness to be “completely blocking”. Thin, compact semiconductor TiO_2_ layer deposited directly on the FTO glass, underneath the mesoporous TiO_2_ layer is also an important part of dye sensitised solar cell (DSSC). Annealing step (450–550 °C, 30–60 min) in fabrication of DSSC is necessary to (i) remove organic binders, (ii) improve interparticle connectivity and (iii) improve crystallinity and phase stability.^10,11^

Beside blocking properties chemical stability of protective overlayers is also important. Recently we have investigated in detail ALD Al_2_O_3_ films and found that these films rapidly dissolved in 1 M NaOH (≈100 nm h^−1^). The dissolution in 1 M H_2_SO_4_ was slower (1 nm h^−1^) but after 24 h the blocking behaviour was entirely lost. The optimal stability was reached at pH 7.2 where no changes were found up to 24 h and even after 168 h of exposure the changes in the blocking behaviour were still minimal.^12^ Wang^13^ reported, that ALD TiO_*x*_ coatings significantly enhanced corrosion resistance and electrical characteristics of titanium proton exchange membrane (PEM) based water electrolyzers.

Several studies^14–17^ investigated influence of ALD process temperature, type of precursors and substrate on mechanism of TiO_2_ growth and crystallinity. For atomic layer deposition of TiO_2_ various precursors exist,^17^ most commonly used are titanium tetrachloride (TiCl_4_), titanium isopropoxide (Ti[OCH(CH_3_)_2_]_4_) and tetrakis(dimethylamino)titanium (TDMAT) in combination with ozone, O_2_ or Ar–O_2_ plasma and H_2_O as oxidants. TDMAT has the advantage that precursor and decomposition products are non-toxic and non-corrosive.^18,19^

The aim of the present study was to look how different ALD deposition procedures using TDMAT and H_2_O as precursors and following annealing step influence morphology of thin TiO_2_ layers, phase composition, and chemical stability in alkaline and acidic pH, as well as their (photo)electrochemical behavior, which are properties essential for their application as protective overlayers.

## Experimental

Atomic layer deposition (ALD) was carried out using a thermal-mode ALD R-200 system (Picosun, Finland) with varying numbers of identical deposition cycles. Two ALD procedures were used for preparation of TiO_2_ films using tetrakis(dimethylamido)titanium(iv) (TDMAT from Strem Chemicals, USA) and water (EpiValence, UK) as precursors with different pulse-purge sequence.^20^ TDMAT was heated to 85 °C, and water was maintained at 22 °C. Nitrogen (99.999%) was used as the carrier gas. The processing temperatures were 150 °C for low temperature TiO_2_ films (LT-ALD TiO_2_) and 250 °C for the high temperature TiO_2_ films (HT-ALD TiO_2_), respectively. Layer thickness was controlled by the number of ALD deposition cycles and determined by ellipsometry^20^ or AFM profile analysis of a step formed on a Si/SiO_2_ substrate following removal of the TiO_2_ film by scratching (Fig. S1 in SI). For post annealing, the films were calcined for 1 h in air at 500 °C, the heating ramp was 10 °C min^−1^. Annealing conditions were selected with aim to form anatase crystalline structure and due to possible application in DSSC. Fluorine-doped tin oxide coated 2 mm thick glass (“FTO”, 7 Ω □^−1^), obtained from Merck (Germany) and an Si/SiO_2_ (300 nm) wafer (Silicon Quest International, USA), were used as substrates. Substrates were cleaned using ethanol, acetone and isopropyl alcohol before deposition procedure.

X-ray photoelectron spectroscopy (XPS) measurements were performed using an ESCA Probe P spectrometer (Omicron Nanotechnology Ltd, Germany) equipped with a monochromatic Al Kα radiation source (1486.7 eV). The measurements were carried out under ultra-high vacuum conditions (base pressure ∼5 × 10^−10^ mbar). To compensate for surface charging during the analysis, a low-energy electron flood gun was employed. For spectral acquisition, a pass energy of 50 eV with a step size of 0.4 eV was used for survey spectra, while high-resolution spectra of C 1s, O 1s, and Ti 2p were recorded with a pass energy of 30 eV and a step size of 0.1 eV. All binding energies were calibrated with respect to the C 1s peak at 284.8 eV. The instrument was calibrated using reference binding energies of Cu 2p_3/2_ (932.7 eV) and Ag 3d_5/2_ (368.26 eV), with respective full width at half maximum values of 0.85 eV and 0.67 eV. Data processing and peak fitting were performed using CasaXPS software (ver. 2.3.17PR1.1), applying Shirley background subtraction and a Gaussian–Lorentzian (GL(30)) peak shape function.

X-ray diffraction (XRD) patterns were recorded using an X'Pert Philips MPD diffractometer (The Netherlands) equipped with a PANalytical X'Celerator detector (PIXcel1D, 1D mode) and operated with Cu Kα_1_ radiation (*λ* = 1.54060 Å) generated at 40 kV and 30 mA. Measurements were performed in continuous scan mode over the 2*θ* range of 5.0–90.0° with a step size of 0.039° and a scan time of 175 s per step. The goniometer radius was 240 mm, and the specimen length was 10 mm. A fixed divergence slit (1.0°) was used, and no spinning or incident beam monochromator was applied. Data analysis and phase identification were carried out using the HighScore Plus software (ver. 5.1.0.29607).

The morphology of the films was characterized by atomic force microscopy (AFM, Dimension Icon, Bruker, USA) in semicontact (tapping TM) or peak force quantitative nanomechanical (PFQNM) mode. A silicon VTESPA-300 cantilever with a resonant frequency, *f*_res_, of approx. 300 kHz, a spring constant, *k*, of 42 N m^−1^, a nominal tip radius of 5 nm (Bruker, USA) and SCANASYST-AIR cantilever, with a resonant frequency, *f*_res_, of approx. 65 kHz, a spring constant, *k*, of 0.4 N m^−1^, and a nominal tip radius of 2 nm (Bruker, USA) were employed for TM and PFQNM, respectively. The Gwyddion software (ver. 2.53) was used for processing AFM image data and for calculation of the roughness factor (*R*_f_), which represents the ratio between the three-dimensional surface area of the image and its two-dimensional footprint area. The Raman spectra were measured by the MicroRaman system (WITec Alpha 300 R spectrometer, Oxford Instruments) with a Confocal microscope. The spectra were excited by a 532 nm excitation laser.

The blocking properties of the deposited layers were evaluated by cyclic voltammetry (CV) in an aqueous electrolyte composed of 0.5 mM K_3_[Fe(CN)_6_] and 0.5 mM K_4_[Fe(CN)_6_] in 0.5 M KCl pH 2.5 (pH value was adjusted by HCl). Electrochemical experiments were carried out in a one-compartment three-electrode cell using Zahner Zennium X workstation (Zahner-Elektrik, Germany) and Autolab PGSTAT 101 potentiostat (Metrohm, The Netherlands) controlled by the NOVA software.

For dissolution studies, TiO_2_ films were exposed to 0.1 M HClO_4_, and 0.1 M phosphate buffer solution (pH 8) for 72 h. Long term stability of film was studied in 1 M NaOH, 1 M H_2_SO_4_ and 0.1 M phosphate buffer (pH 7).

The determination of the titanium concentration in solution was carried out by inductively coupled plasma spectrometry (ICP-OES) using an Optima 8000 instrument (PerkinElmer, USA).

For photoelectrochemical measurements of the incident photon to current conversion efficiency (IPCE) spectra, an Electrochemical Photocurrent Spectra CIMPS-pcs system (Zahner-Elektrik, Germany) with a TLS03 tunable light source was used. The three-electrode system with the TiO_2_-coated FTO working electrode, Ag/AgCl (3 M KCl) reference electrode and platinum rod as a counter electrode in 0.1 M Na_2_SO_4_ (pH 10) electrolyte solution was used for photoelectrochemical experiments. The TiO_2_ films were illuminated from the front side (EE interface).

## Results and discussion

### Physical characterization

**Fig. 1:**
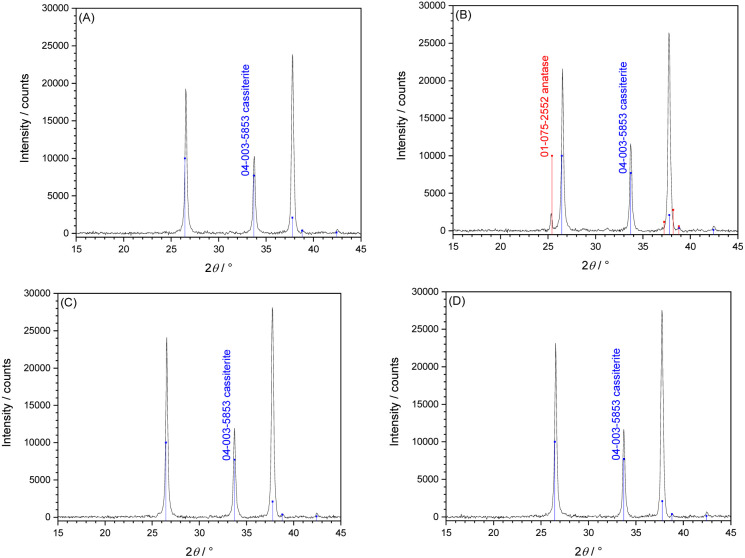
XRD of 50 nm TiO_2_ (ALD) layers on FTO/glass, (A) and (C) as-deposited, (B) and (D) annealed at 500 °C for 1 h in air. Deposition at 150 °C LT-ALD (A and B) and 250 °C HT-ALD (C and D). XRD reference lines: 04-003-5853 (cassiterite) and 01-075-2552 (anatase).^22^

Two types of TiO_2_ thin films were deposited by Atomic layer Deposition (ALD), at lower deposition temperature 150 °C (LT-ALD TiO_2_) and higher deposition temperature 250 °C (HT-ALD TiO_2_). Two different ALD procedures were selected based on the initial assumption that deposition at 150 °C formed amorphous^17^ TiO_2_, whereas the crystalline form^16^ of TiO_2_ can be obtained at process temperature 250 °C. Fig. 1 shows the X-ray diffractograms of ALD TiO_2_ films, where cassiterite lines originated from FTO substrate. XRD confirms that as-deposited LT-ALD TiO_2_ film is amorphous, but annealing at 500 °C/1 h in air causes crystallization towards anatase (Fig. 1A and B). The intensity of the strongest anatase diffraction line does not increase after annealing at 500 °C for 2 h, indicating that 1 h of annealing is sufficient for complete anatase formation in the TiO_2_ film. Although Chiappim *et al.*^16^ reports the formation of anatase crystalline phase at ALD process temperature in the range 250–300 °C, the XRD (Fig. 1C and D) indicates that HT-ALD deposition method did not result in the formation of any crystalline phase, even after post-annealing at 500 °C. The poor crystallization tendency of TiO_2_ films deposited by ALD using alkylamide-based precursors at processing temperature 200–260 °C was also previously reported.^17^ The crystallization of the TiO_2_ films was found to be dependent on film thickness and impurity content, but effect of impurities on the crystallization behavior could only be observed for very thin films (<30 nm).^21^ Abendorth^18^ reported, that TiO_2_ crystallization temperature was also dependent on H_2_O purge times. The plausible explanation of the finding, that HT-ALD deposition method leads to formation of amorphous TiO_2_ even after post-annealing at 500 °C remains so far unknown and is not within the scope of this paper.

**Fig. 2:**
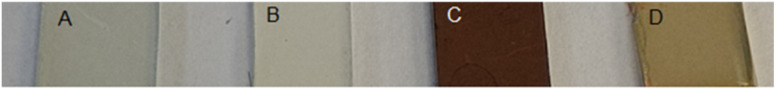
Photography of as-deposited (A and C) and annealed (B and D) 50 nm TiO_2_ layers on FTO support. (A) As-deposited LT-ALD TiO_2_; (B) annealed LT-ALD TiO_2_; (C) as-deposited HT-ALD TiO_2_; (D) annealed HT-ALD TiO_2_.

Fig. 2 shows photo images of 50 nm TiO_2_/FTO electrodes. While as deposited and annealed LT-ALD TiO_2_ films are rather transparent (Fig. 2A and B), as-deposited HT-ALD TiO_2_ has dark brown color (Fig. 2C), which is typical for reductively doped TiO_2_.^23–25^

**Fig. 3:**
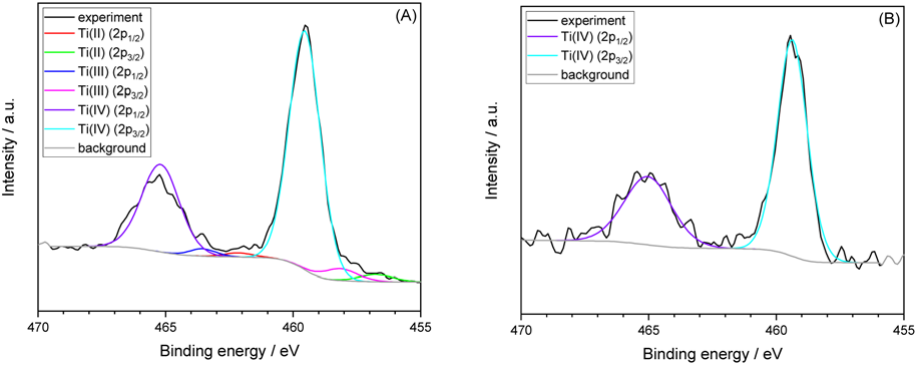
XPS spectra of HT-TiO_2_ as deposited (A) and after annealing at 500 °C/1 h (B). The envelope curve is not included in the figures, as it significantly overlapped with the fitted components and made their individual contributions indistinguishable.

XPS spectra of HT-TiO_2_ as-deposited and after annealing at 500 °C/1 h are shown in Fig. 3. We can see that XPS analysis confirmed self-doped sites with oxygen vacancies such as Ti^3+^ (around 5%) and other low-valent Ti (around 2% of Ti^2+^) in as-deposited film (Fig. 3A). Therefore, HT-ALD deposition method forms non-stochiometric TiO_2−*x*_. The presence of Ti^3+^ in ALD grown amorphous TiO_2_ was reported by Saari *et al.*^26^ The decrease of oxygen to titanium ratio ([O]/[Ti]) with increasing deposition temperature was also observed by Kim *et al.*^15^ Annealing at 500 °C/1 h in air causes thermal re-oxidation of Ti(iii) and Ti(ii) to Ti(iv), which is confirmed by XPS analysis (Fig. 3B). The decrease in the level of doping is indicated by the change of HT-ALD TiO_2_ film color from brown to yellowish (Fig. 2D).

Nanomorphology of TiO_2_/FTO films was characterized by AFM (Fig. S2 in SI). Both as deposited and annealed samples do not show significant difference in nanomorphology and roughness factor. Their nanomorphology however differs from bare FTO support (Fig. S2E in SI), clearly indicating presence of deposited TiO_2_ films. Table S1 (in SI) summarizes roughness factors for as-deposited LT- ALD TiO_2_ films at the FTO substrate for varying film thicknesses between 8 and 50 nm. There is no significant difference in *R*_f_ compared to bare FTO, but for 50 nm thick TiO_2_ film *R*_f_ slightly decreases. We can assume conformal coverage of FTO for all our TiO_2_ films, which is also evidenced by AFM height density distributions (Fig. S3 in SI).

**Fig. 4:**
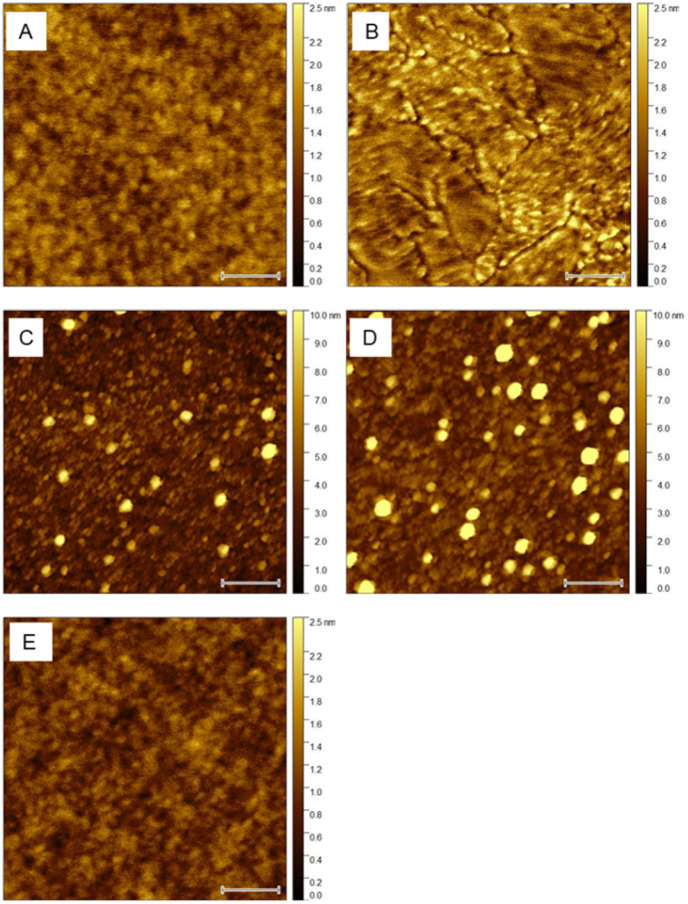
AFM height images (0.5 µm × 0.5 µm) of 8 nm TiO_2_ layers on SiO_2_/Si support – as deposited (A and C) and annealed (B and D). (A) As deposited LT-ALD TiO_2_, *R*_f_ = 1.03; (B) annealed LT-ALD TiO_2_, *R*_f_ = 1.03; (C) as deposited HT-ALD TiO_2_, *R*_f_ = 1.10; (D) annealed HT-ALD TiO_2_, *R*_f_ = 1.10; (E) bare SiO_2_/Si *R*_f_ = 1.02. White bars represent 100 nm.

As the FTO support has quite rough surface, the atomic force microscopy resolution is rather limited. Consequently, the application of well-defined flat substrate, such Si/SiO_2_ wafer is needed for detailed high-resolution study of nanomorphology of ALD-TiO_2_ thin film (Fig. 4). Apparently as deposited LT-TiO_2_ films (Fig. 4A) are amorphous, conformal to SiO_2_ nanomorphology (Fig. 4E), whereas annealed LT-TiO_2_ films form crystalline nanodomains with visible boundaries (Fig. 4B). Roughness factor *R*_f_ of HT-ALD TiO_2_ films (*R*_f_ = 1.10) is higher compared to LT-ALD TiO_2_ films (*R*_f_ = 1.03). Nanomorphology of as-deposited HT-ALD TiO_2_ film (Fig. 4C) did not significantly change after post-annealing (500 °C/1 h) procedure (Fig. 4D).

Dissipation AFM mode based on energy loss during tip–sample interaction is mapping surface nanomechanical properties beyond simple topography and therefore it is used to clarify presence/absence of surface grain-boundary structures independently on the surface roughness (Fig. S4). While surface of annealed LT-ALD TiO_2_ (Fig. S4A) is composed from crystalline grains of different orientation with clearly recognized boundaries, the surface of annealed HT-ALD TiO_2_ (Fig. S4B) shows no such grain-boundary nanomorphology.

### Electrochemical barrier properties of ALD films deposited on FTO

**Fig. 5:**
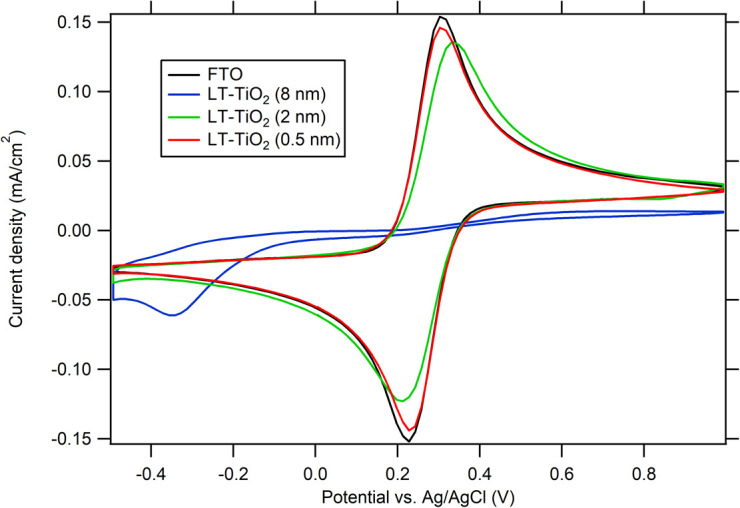
CVs of 0.5 mM K_3_[Fe(CN)_6_] and 0.5 mM K_4_[Fe(CN)_6_] in 0.5 M KCl on FTO electrodes covered with as-deposited LT-ALD TiO_2_ films with thickness values of 0.5, 2 and 8 nm, respectively. The scan rate was 50 mV s^−1^.

Redox system [Fe(CN)_6_]^3−/4−^ was used for testing the electrochemical blocking properties of ALD TiO_2_/FTO films. The [Fe(CN)_6_]^3−/4−^ redox couple was chosen as a simple one-electron transfer-reaction probe with redox potential positive to flatband of TiO_2_. Thus titanium dioxide behaves like an electrochemically silent dielectric material against the [Fe(CN)_6_]^3−/4−^ couple and the charge–transfer reaction is assumed to occur solely at FTO surface directly exposed to the electrolyte solution.^27^ While the blocking of the [Fe(CN)_6_]^3−/4−^ redox reaction (decrease in the peak height) was observed for 8 nm LT-ALD TiO_2_, for 0.5 and 2 nm thick LT-ALD TiO_2_ films the oxidation peak of [Fe(CN)_6_]^4−^ was the same as for bare FTO. The TiO_2_ layers were too thin and their barrier properties were not sufficient (Fig. 5).

For the evaluation of the FTO surface fraction covered by the titania blocking layer the method described previously by Kavan *et al.*^27^ was used. The effective pinhole area (EPA_EC_) can be expressed by the eqn (1).

1 EPA_EC_ = *A*_uc_/*A*_0_ = *j*_p_/*j*_p,FTO_ × 100 (%)

where *A*_uc_ is the uncovered area of FTO and *j*_p,FTO_ is the peak current density measured at a bare FTO electrode; *j*_p_ is the peak current density measured at the TiO_2_/FTO electrode and *A*_0_ is projected electrode area. The blocking characteristics (EPA_EC_) are summarized in Table 1.

**Table 1:** The blocking properties ALD TiO_2_ layers on FTO

	As deposited		Annealed	
	EPA_EC_/%	Defect type	EPA_EC_/%	Defect type
8 nm LT-TiO_2_	7	B	43	A/B
20 nm LT-TiO_2_	6	B	18	B
50 nm LT-TiO_2_	1	B	2	B
8 nm HT-TiO_2_	—	—	17	A/B
20 nm HT-TiO_2_	—	—	5	B
50 nm HT-TiO_2_	—	—	1	B

As suggested in work^27^ there are two types of defects in the barrier film: The “defect A”, in which the partially blocked electrode behaves like “clean” FTO, but with a relatively smaller effective area. The relative increase of the voltammetric peak separation Δ*E*_pp_ < 3 (Δ*E*_pp_ is defined as the difference between the peak potential values for the Fe(CN)_6_^4−^ oxidation and Fe(CN)_6_^3−^ reduction on the blocking layer, normalized to that on pure FTO). The “defect B” is a more complex situation, in which the defect not only causes the delamination of the titanium dioxide film from the FTO substrate, but also the slowdown of charge transfer kinetics (accompanied by a strong increase in Δ*E*_pp_).

**Fig. 6:**
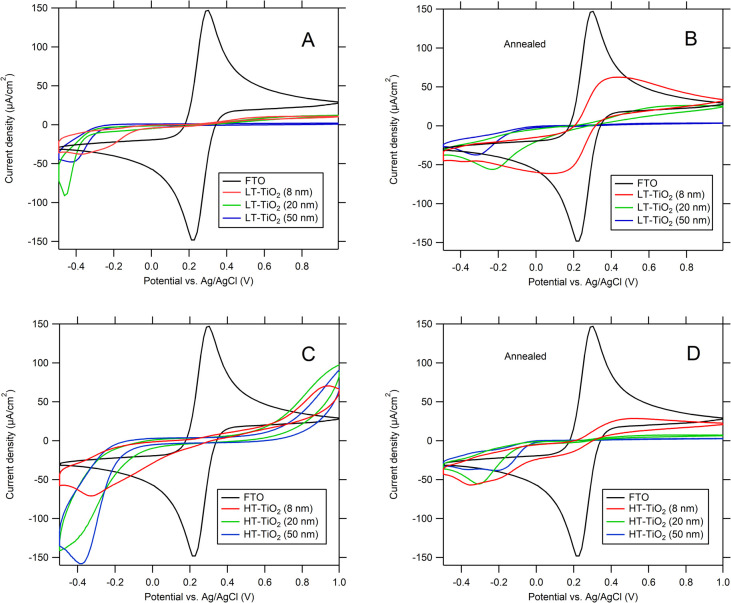
Influence of film thickness on the blocking properties of ALD-TiO_2_ films as-deposited (A and C), and after annealing (500 °C/1 h) (B and D). LT – ALD TiO_2_ (A and B); HT – ALD TiO_2_ (C and D); electrolyte 0.5 mM K_3_[Fe(CN)_6_] and 0.5 mM K_4_[Fe(CN)_6_] in 0.5 M KCl. The scan rate was 50 mV s^−1^.

Blocking properties of as-deposited ALD TiO_2_ layers and after annealing (layer thickness 8 nm, 20 nm and 50 nm) are shown in Fig. 6. As-deposited LT-ALD TiO_2_ layers of thickness 8–50 nm FTO blocked well electrochemical reaction of [Fe(CN)_6_]^3−/4−^ (Fig. 6A), increasing thickness decrease the calculated value of EPA_EC_. The annealing decreased its barrier properties (Fig. 6B) due to rearrangement of TiO_2_ to crystalline nanograins (Fig. 4B and S4A), which boundaries may allow leakage of electrolyte to FTO support. Then, FTO support covered by TiO_2_ film may acts as array of microlectrodes, where spherical diffusion may affect the shape of voltammetric curves (Fig. 6). Still, the large (>>59 mV) potential separation of anodic and cathodic redox current maxima and pronounced peak shoulders indicate also slow kinetics with iR drop contribution, which both affect the EPA_EC_ estimation with an error rising with thickness of TiO_2_ deposit blocking layer.

In the case of as-deposited HT-ALD TiO_2_, we cannot distinguish between redox reaction taking place at conductive FTO and self-doped HT-ALD TiO_2_ (Fig. 6C), thus the determination of pinhole area by this method is not applicable. An alternative electrochemical barrier evaluation can be performed using the electrochemical reaction of a substrate other than bare FTO that has a specific electrochemical response, for example, an Au substrate. However, a disadvantage of this technique—besides the high cost of Au substrates—is the relatively high mobility of Au, which can alter the performance of ALD films, especially after annealing, due to Au contamination.^28,29^

After re-oxidation of self-doped HT-TiO_2_ layer by annealing, the determination of its electrochemical blocking properties become feasible (Fig. 6D). Nevertheless, the cyclic voltammetry yields peaks with shoulders, less pronounced current maxima, and large peak separation, which indicate only slow reaction kinetics, because absence of grain boundaries (Fig. S4B) excluded microelectrode effect.

### Chemical stability

Chemical stability of TiO_2_ films deposited on FTO support in 0.1 M HClO_4_ (pH 1) and 0.1 M phosphate buffer (pH 8) was evaluated from dissolution rates in these solutions (Table 2). The samples were exposed to different electrolytes for 72 hours in dark. The dissolution rates (nm h^−1^) of TiO_2_ were calculated from concentrations of titanium dissolved in electrolytes determined by ICP analysis, using the geometric area of exposed TiO_2_ films (2 cm^2^), the volume of the electrolyte (20 ml) and the density of TiO_2_ (*ρ*_amorphous_ = 3.59 g cm^−3^ for as-deposited TiO_2_ films and annealed HT-ALD TiO_2_, *ρ*_anatase_ = 3.77 g cm^−3^ for annealed LT-ALD TiO_2_ (ref. 16)) neglecting porosity of the films and assuming uniform coverage (Table 2). In acidic solution more than 40% LT-ALD and 60% HT-ALD of TiO_2_ thickness (*z*) was dissolved after 72 h exposure. Post annealing (500 °C/1 h) caused mild improvement of chemical stability for both LT-ALD and HT-ALD TiO_2_.

**Table 2:** Dissolution rates of 8 nm TiO_2_ films determined by ICP-OES analysis^a^

Sample	Solution	*z*/nm	Dissolution rate/nm h^−1^
As dep. LT- TiO_2_	0.1 M HClO_4_	3.83 ± 0.38	0.053 ± 0.005
Anneal. LT-TiO_2_		2.69 ± 0.20	0.038 ± 0.003
As dep LT- TiO_2_	0.1 M PB (pH 8)	0.28 ± 0.03	0.004 ± 0.001
Anneal. LT-TiO_2_		0	0
As dep. HT- TiO_2_	0.1 M HClO_4_	5.25 ± 0.57	0.073 ± 0.008
Anneal. HT-TiO_2_		4.44 ± 0.04	0.061 ± 0.001
As dep. HT- TiO_2_	0.1 M PB (pH 8)	0.61 ± 0.11	0.009 ± 0.002
Anneal. HT-TiO_2_		0	0

a *z* concentration of dissolved titanium recalculated to thickness of dense compact TiO_2_ layer; PB phosphate buffer.

**Fig. 7:**
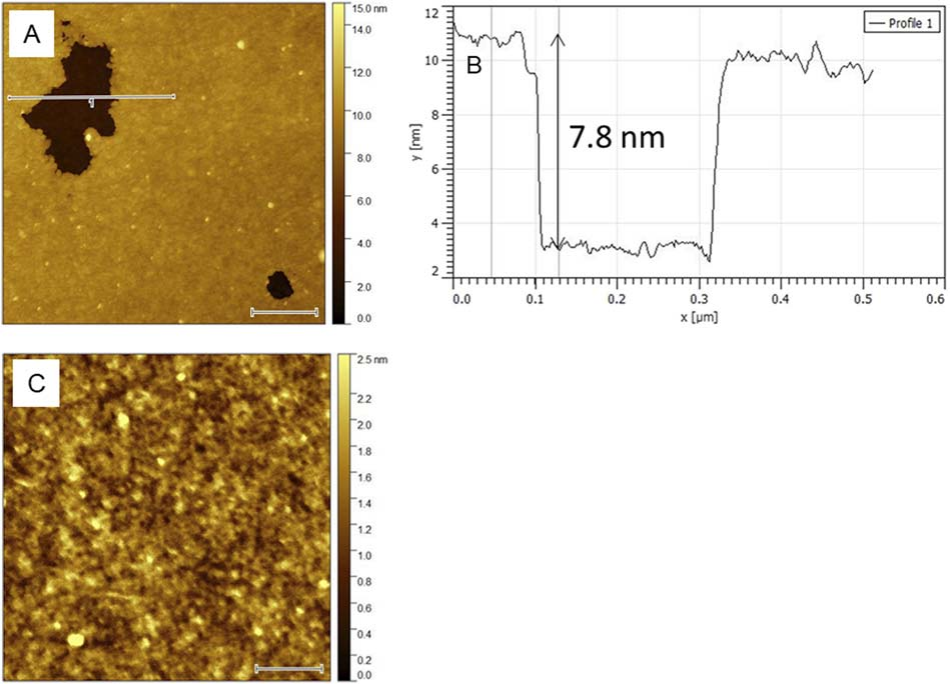
AFM of as-deposited 8 nm LT-ALD TiO_2_ on SiO_2_/Si support after 72 h dissolution in 0.1 M HClO_4_ pH 1. (A) AFM height image 1 µm × 1 µm, white bar represents 200 nm, line 1 demonstrates pinhole profile analysis (B); (C) AFM height image 0.5 µm × 0.5 µm, surface nanomorphology of TiO_2_ outside the pinholes, white bar represents 100 nm.

The etching of both as-deposited and annealed LT-ALD TiO_2_ films by exposing to acidic solution led to the formation of pinholes with submicron area and depth 7.6 ± 0.3 nm determined from AFM profile analysis of all pinholes appearing in examined areas (Fig. 7A, B and S5A–S5B). This value corresponds to the thickness (≈8 nm) of deposited TiO_2_. Surface nanomorphology after etching (Fig. 7C and S5C in SI) was identical (except pinholes) to the nanomorphology of original LT-ALD TiO_2_ film (Fig. 4).

Based on intact TiO_2_ nanomorphology after exposure to acidic media (Fig. 7 and S5 in SI) we can assume, that dissolution occurs only locally, forming pinholes. On this basis, we recalculated the amount of dissolved titanium, determined by ICP-OES analysis, to the area, which would be uniformly covered by dense, 8 nm thick TiO_2_ film. This area (A_ph_), corresponding to sum of areas of all pinholes, was normalized to total surface area (*A*_tot_ = 2 cm^2^) of the sample. This ratio (eqn (2)) thus represents relative increase in effective pinhole area after the exposure to acidic media. While EPA_EC_ was determined from electrochemical barrier properties (see Section 3.2), the value obtained from ICP results is denoted as ΔEPA_ICP_ (Eqn (2)). The error of the ΔEPA_ICP_ determination (max. 10%) is governed by the fluctuation of amount of dissolved titania (Table 2).

2 ΔEPA_ICP_ = *A*_ph_/*A*_tot_ × 100 (%)

**Fig. 8:**
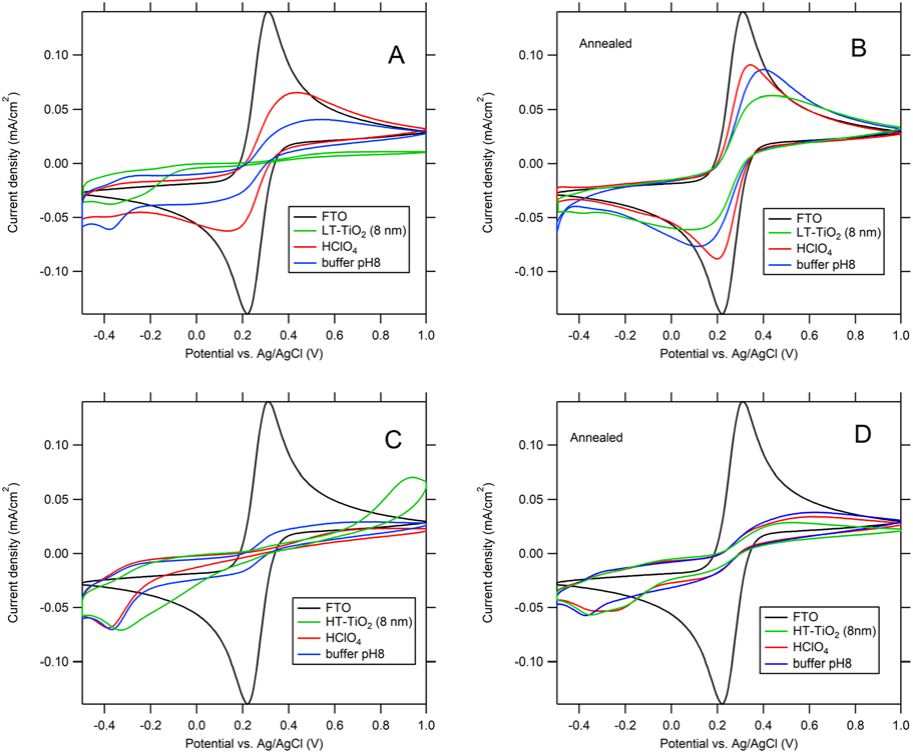
The blocking properties of 8 nm ALD TiO_2_ layers as deposited (A and C) and annealed (B and D) on FTO after 72 h exposure to 0.1 M HClO_4_ pH = 1, and 0.1 M phosphate buffer pH = 8. (A and B) LT-ALD TiO_2_; (C and D) HT- ALD TiO_2_. The scan rate was 50 mV s^−1^.

Fig. 8 shows, that electrochemical blocking properties of 8 nm TiO_2_/FTO electrodes after 72 h exposure to different solution (pH 1 and 8) decreased for all studied TiO_2_ layers. The shape of voltammetric curves (red curves in Fig. 8A and B) of electrode with pinholes etched in TiO_2_ layer indicates slower kinetics rather than involvement of microelectrode effect.

**Fig. 9:**
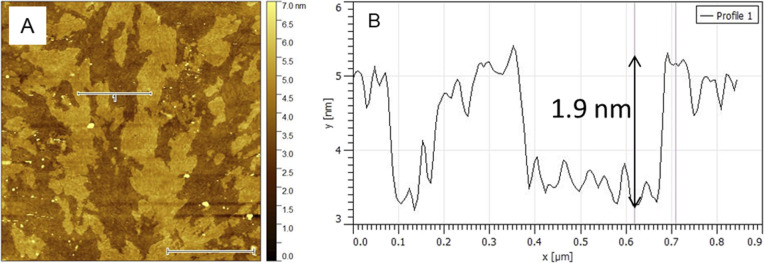
AFM of annealed 8 nm LT-ALD TiO_2_ on SiO_2_/Si support after 72 h dissolution in 0.1 M phosphate buffer pH 8. (A) AFM height image (white bar represents 1 µm), line 1 shows location of profile analysis (B).

Difference of EPA evaluated from current peak density of redox reaction of ferro/ferricyanide on TiO_2_/FTO electrodes before and after dissolution experiment (Table S2) is denoted as ΔEPA_EC._Table 3 correlates values of ΔEPA_ICP_ with ΔEPA_EC_. While both methods show good agreement for LT-ALD TiO_2_ exposed to an acidic environment, determining of chemical stability in an alkaline environment is likely more complicated. Although the ICP-OES analysis indicated the dissolution of less than 4% of LT-ALD TiO_2_ (Table 2), its electrochemical blocking properties after 72 h exposure to phosphate buffer (pH 8) significantly decreased (Fig. 8A, B and Table 3). This discrepancy can be explained by adsorption of phosphate on TiO_2_,^30,31^ and formation of several types of Ti-phosphate complexes such as TiO(OH)(H_2_PO_4_)·2H_2_O and *etc.*,^31^ which decreased amount of dissolved titanium in solution available for ICP-OES analysis and influenced electrochemical blocking properties of TiO_2_ layer. Presence of ∼2 nm thick layer on annealed LT-ALD TiO_2_ observed by AFM (Fig. 9A and B) after exposition to phosphate buffer (pH 8), appears to confirm formation of adsorbed layer on TiO_2_ surface. No pinholes were observed in examined areas after exposure to phosphate buffer pH 8.

**Table 3:** A relative increase in effective pinhole area (EPA) in 8 nm TiO_2_ film after 72 h dissolution in 0.1 M HClO_4_ pH = 1 and 0.1 M phosphate buffer pH = 8, calculated from chemical (ICP) and electrochemical analysis (EC)

Sample	ΔEPA_ICP_ (%)	ΔEPA_EC_ (%)	
	pH 1	pH 1	pH 8
LT-ALD TiO_2_	48	40	22
LT-ALD TiO_2_ annealed	34	22	19
HT-ALD TiO_2_	65	—	—
HT-ALD TiO_2_ annealed	56	7	10

In the case of as-deposited HT-ALD TiO_2_, mentioned above, determination of barrier properties using electrochemical model redox couple cannot be used due to presence of self-doped sites. For annealed HT-ALD TiO_2,_ the XPS analysis did not detect presence of low valence titanium (the concentration of Ti^3+^ may be below the detection limit of XPS, which is only 0.5 at%),^32^ but the large differences between the ΔEPA_ICP_ and values ΔEPA_EC_ (Table 3), together with the yellowish color of annealed HT-ALD TiO_2_ films (Fig. 2D), typical for nonstoichiometric TiO_2_,^25^ indicate, that the electrochemical method of determination pinholes area has limited validity also for annealed HT-ALD TiO_2_ electrodes.

Long-term stability (168 h) was tested for 8 nm as-deposited LT-ALD TiO_2_/FTO. Fig. S6 (in SI) shows the electrochemical blocking properties of LT-TiO_2_/FTO electrode after dissolution in 1 M NaOH, 1 M H_2_SO_4_ and neutral phosphate buffer. After 168 hours exposure to sulphuric acid 8 nm LT-ALD TiO_2_ was totally decomposed, effective pinhole area reached 99%, while exposure to solutions of neutral and alkaline pH led to EPA increase to 13% and 27% respectively (Fig. S6 in SI).

### Photoelectrochemistry

**Fig. 10:**
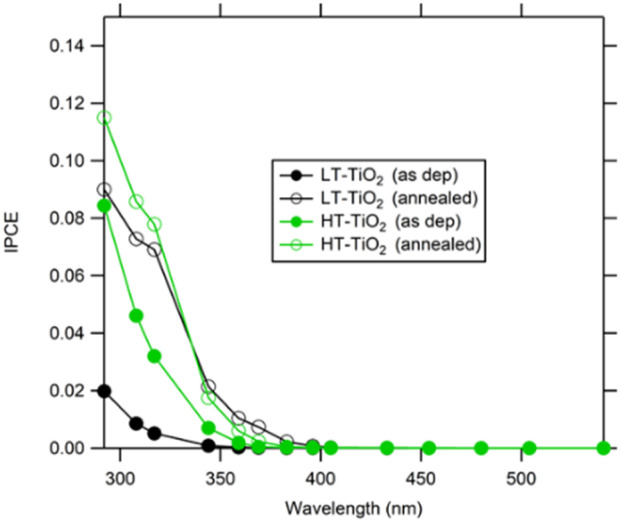
IPCE spectra of 8 nm TiO_2_/FTO, electrolyte 0.1 M Na_2_SO_4_, pH 10, potential 1 V *vs.* Ag/AgCl, illumination geometry EE. LT-ALD TiO_2_ – black circles; HT-ALD TiO_2_ – green circles. Filled circles – as-deposited TiO_2_; open circles – annealed TiO_2_.

Fig. 10 shows the incident photon to current conversion efficiency (IPCE) spectra for both types of ALD films measured at applied potential 1 V *vs.* Ag/AgCl. Fig. S7 then shows photocurrent for irradiation at 369 nm (where IPCE values are very small) as a function of time. As-deposited HT-ALD TiO_2_ films exhibit significantly higher (4 times) IPCE values compared to as-deposited LT-ALD TiO_2_, which can be explained by the self-doping.^25,33,34^ For as-deposited HT-ALD TiO_2_ the presence of low valence titanium introduce electronic states within the bandgap of TiO_2_ and caused the shift of absorption edge to longer wavelength.^33^ Several recent studies have shown that amorphous or disordered materials can display higher photocatalytic activity than their crystalline counterparts. *E.g.* Zywitzki^35^ reports amorphous TiO_2_ with Ti^3+^ which has blue color and is active in photocatalytic H_2_ generation. Enhancement of photoactivity due to the presence of Ti^3+^ but in crystalline TiO_2_ was also reported.^36–38^

Annealing of LT-ALD TiO_2_ films caused significant (4–5 times) increase of IPCE, this can be explained by the formation of anatase crystalline structure. An increase in the photocurrent at 369 nm is even higher (20–40 times). Annealing of HT-ALD TiO_2_ films results also in an increase in IPCE but in much smaller extent (about 2 times), similar increase was observed for photocurrent at 369 nm. The observed increase in photoelectrochemical response for HT-ALD TiO_2_ films is in contradiction with findings that after annealing in air films remain amorphous (Fig. 1D) and according XPS low valent Ti disappears (Fig. 3B). This can be explained by the two facts. First, low valence Ti are present but below the detection limit of XPS analysis. The presence of small amount of low valence Ti is in agreement with the remaining yellowish color of the HT-ALD film after annealing (see Fig. 2D). Similar observation was reported by Wierzbicka *et al.*^37^ where even small amount of Ti^3+^, not detected by XPS strongly influenced photocatalytic hydrogen evolution. Second, even XRD did not show any crystalline phase, Raman analysis (Fig. S8 in SI) shows the formation of small amount of rutile.

The band gap energy (*E*_g_) was determined from the electrochemical Tauc plot (Fig. S9 in SI). The Tauc – function (indirect transition) was taken as (ln(1/(1–IPCE)) × *hν*)^1/2^.^39^ The calculated value of *E*_g_ is 3.4 and 3.35 eV for as-deposited LT-ALD TiO_2_ and HT-ALD TiO_2_, respectively. After annealing, the band gap of LT- ALD TiO_2_ decreased to 3.1 eV due to the formation of anatase crystalline structure. But for annealed HT- ALD TiO_2_ electrodes band gap did not significantly change, which is in agreement with the observed predominant amorphous structure.

## Conclusions

Parameters of ALD deposition procedure significantly influence properties of deposited TiO_2_ thin films. As-grown TiO_2_ films prepared by ALD with deposition temperature 150 °C (LT-ALD) were amorphous, subsequent annealing at 500 °C/1 h led to the formation of anatase crystalline structure indicated by XRD and also manifested by nanomorphology rearrangement. Formation of crystalline nanodomains with visible boundaries was confirmed by AFM. ALD with deposition temperature 250 °C (HT-ALD) forms amorphous non-stochiometric TiO_2−*x*_ with presence of low valence titanium. Annealing at 500 °C/1 h in air caused the decrease in the level of doping, but no crystalline structure was detected.

As-deposited LT-ALD TiO_2_ layers of thickness 8–50 nm blocked well electrochemical reaction of model redox couple [Fe(CN)_6_]^3−/4−^, calculated EPA was low, but for determination of barrier properties of HT-ALD TiO_2_, this method is not applicable, due to presence of self-doped sites.

ALD TiO_2_ thin films were unstable in acidic solution, exposure to 0.1 M HClO_4_ for 72 h led to the formation of pinholes in TiO_2_ film with submicron area and depth corresponding to TiO_2_ thickness and dissolution of ∼50% and 40% of TiO_2_ of as-deposited HT-ALD TiO_2_ and LT-ALD TiO_2_, respectively. While all tested ALD TiO_2_ films showed good chemical stability in alkaline phosphate buffer (pH 8), AFM indicated formation of thin layer on TiO_2_ surface, which may influence (photo)electrochemical properties of TiO_2_ electrodes. Photoelectrochemical (PEC) response of as deposited LT-ALD TiO_2_/FTO electrode is negligible and significantly increases (about 1 order) due to formation of anatase sructure after annealing. Higher PEC response of as deposited HT-ALD TiO_2_/FTO electrode compared to as-deposited LT- ALD TiO_2_/FTO is caused by Ti^3+^ self-doping. PEC response after annealing of HT-ALD TiO_2_/FTO increased even the amount of Ti^3+^ decreased and XRD did not confirm any crystalline phase. This could be explained by the presence of traces of low valent Ti and/or by the formation of small amount of rutile.

## Author contributions

Hana Krýsová: layer deposition, electrochemical characterization, reviewing and editing, Tomáš Imrich: investigation, XRD analysis, chemical stability, Hana Tarábková: AFM analysis, writing – original draft preparation, reviewing and editing, Pavel Janda: AFM analysis, reviewing and editing, Josef Krýsa: conceptualization, writing, reviewing and editing.

## Conflicts of interest

There are no conflicts to declare.

## Supplementary Material

RA-016-D5RA09703G-s001

## Data Availability

The data presented in this study are available at https://doi.org/10.5281/zenodo.17829888. Data set for “Impact of variations in ALD procedure on nanomorphology, protecting properties and chemical stability of thin TiO_2_ Films” (Original data) (Zenodo). Supplementary information (SI) is available. See DOI: https://doi.org/10.1039/d5ra09703g.
