# Supplementary material for: Impact of variations in ALD procedure on nanomorphology, protecting properties and chemical stability of thin TiO2 films
Source: RSC Adv. 2026 Feb 12;16(10):8658–66. doi: 10.1039/d5ra09703g (PMC12895400; doi:10.1039/d5ra09703g)
Supplement: RA-016-D5RA09703G-s001 [file RA-016-D5RA09703G-s001.pdf]

Supplementary Information (SI) for RSC Advances.
This journal is © The Royal Society of Chemistry 2026

Supporting Information

Impact of variations in ALD procedure on nanomorphology, protecting properties and chemical stability of thin TiO_2 Films

Hana Krýsová[a], Tomáš Imrich[b], Hana Tarábková[a*], Pavel Janda[a] and Josef Krýsa[b*]

[a]J. Heyrovský Institute of Physical Chemistry of the Czech Academy of Sciences, Dolejškova 2155/3, 182 23, Prague 8, Czech Republic

[b]Department of Inorganic Technology, University of Chemistry and Technology, Technická 1905/5, 166 28 Prague 6, Czech Republic

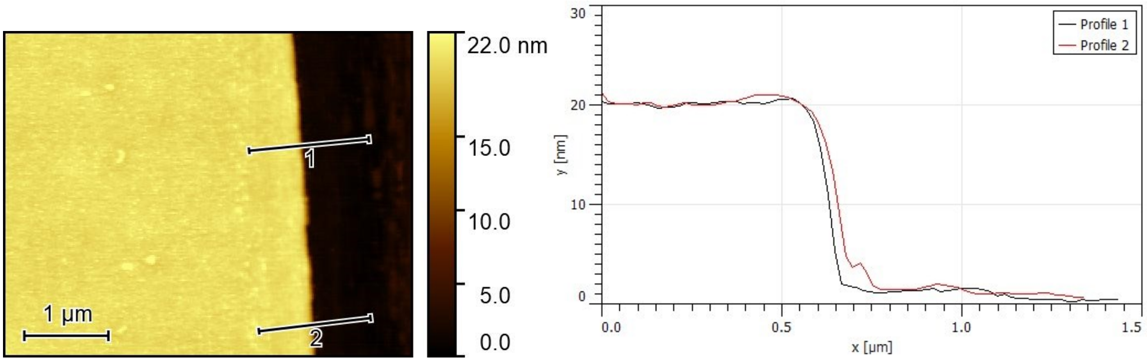

Figure S1. AFM profile analysis of a step formed on a Si/SiO_2 substrate by scratching the HT-ALD TiO_2 film. Two profile lines show average TiO_2-layer thickness 20 nm.

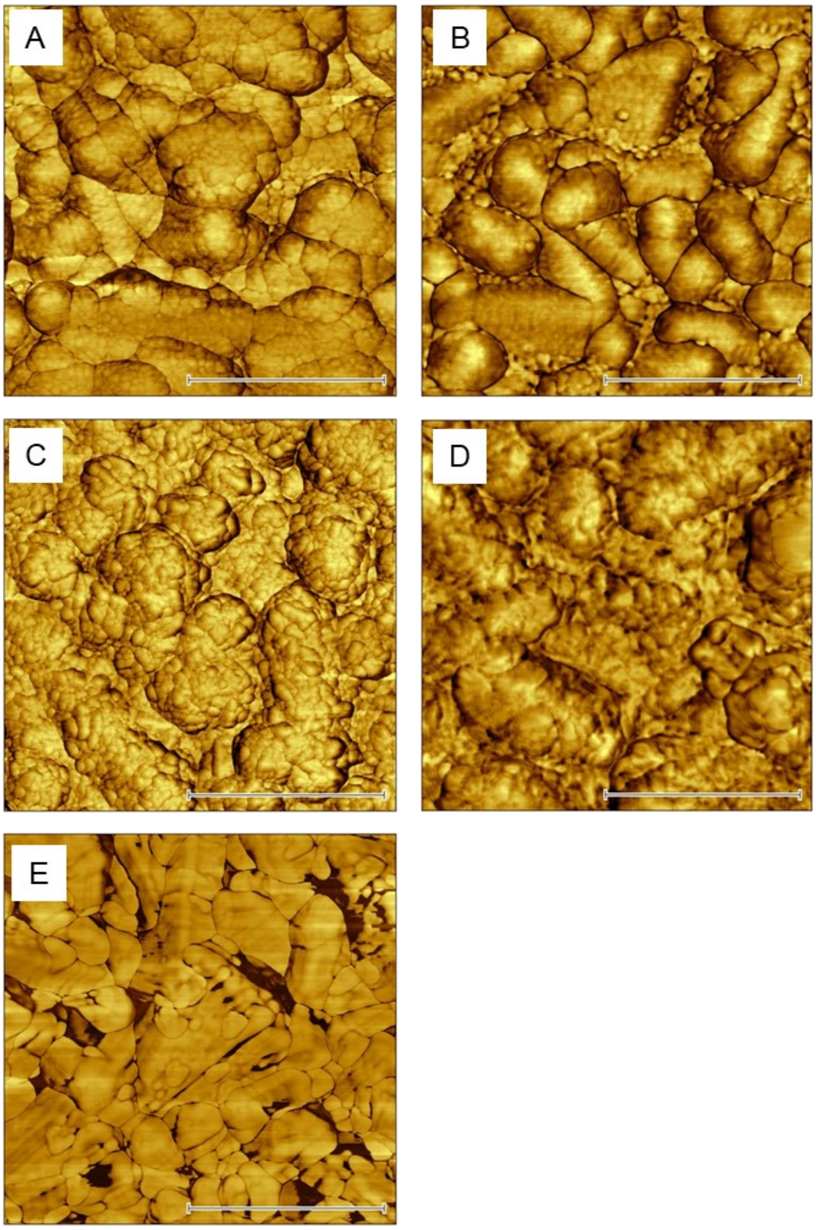

Figure S2. AFM phase images (1 μm × 1 μm) of 50 nm TiO_2 layers on FTO support – as deposited (A, C) and annealed (B, D) for 500 °C/1 h. (A) as deposited LT-ALD TiO_2, R_f = 1.17; (B) annealed LT-ALD TiO_2, R_f = 1.17; (C) as deposited HT-ALD TiO_2, R_f = 1.19; (D) annealed HT-ALD TiO_2, R_f = 1.17; (E) bare FTO. Black bars represent 500 nm. Axial axis shade coded phase shift (degree).

Table S1. R_f of LT-TiO_2 ALD samples as deposited for different TiO_2 thickness, R_f calculated from (5 µm × 5 µm) AFM height images from several locations.

**TiO_2 thickness**	**R_f**
8 nm	1.20±0.02
20 nm	1.22±0.03
50 nm	1.17±0.03
Bare FTO	1.25±0.05

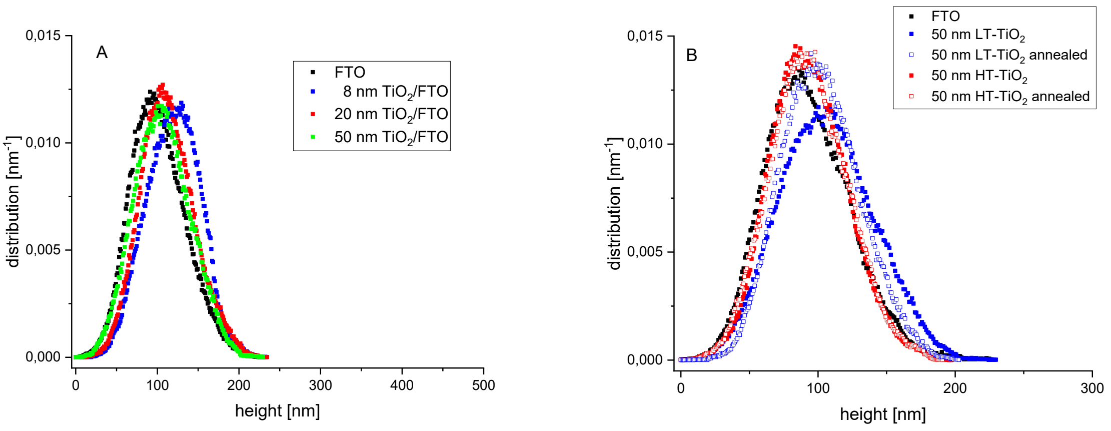

Figure S3. Height density distribution (HDD) calculated from AFM height images (5 µm × 5 µm) of TiO_2/FTO layer. A) HDD for as-deposited LT-ALD TiO_2 with varying thickness, B) HDD for 50 nm TiO_2/FTO prepared by different procedures.

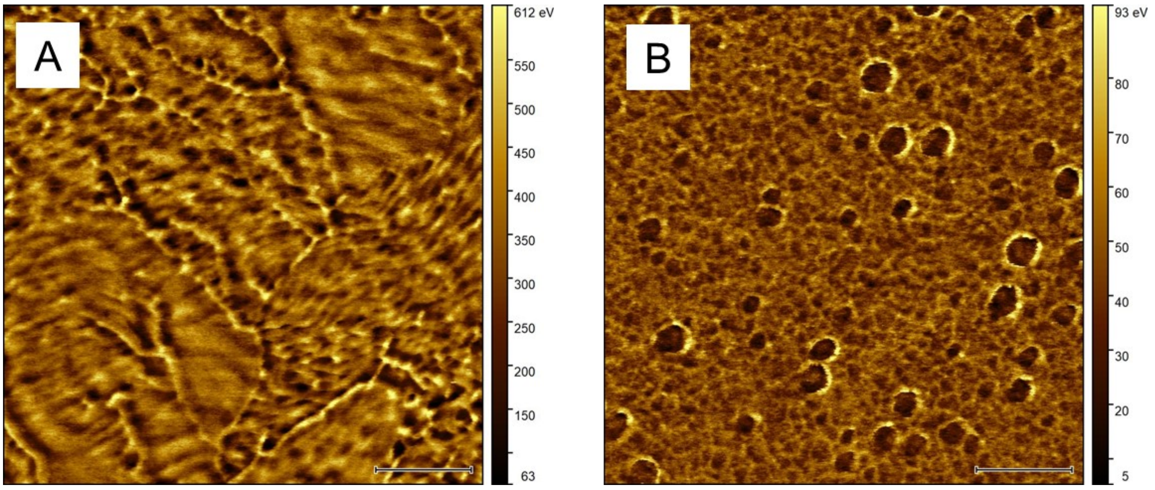

Figure S4. AFM dissipation mode image (0.5 µm × 0.5 µm) of 8 nm TiO_2 deposited on SiO_2/Si support. A) annealed LT-ALD TiO_2; B) annealed HT-ALD TiO_2, black bars represent 100 nm.

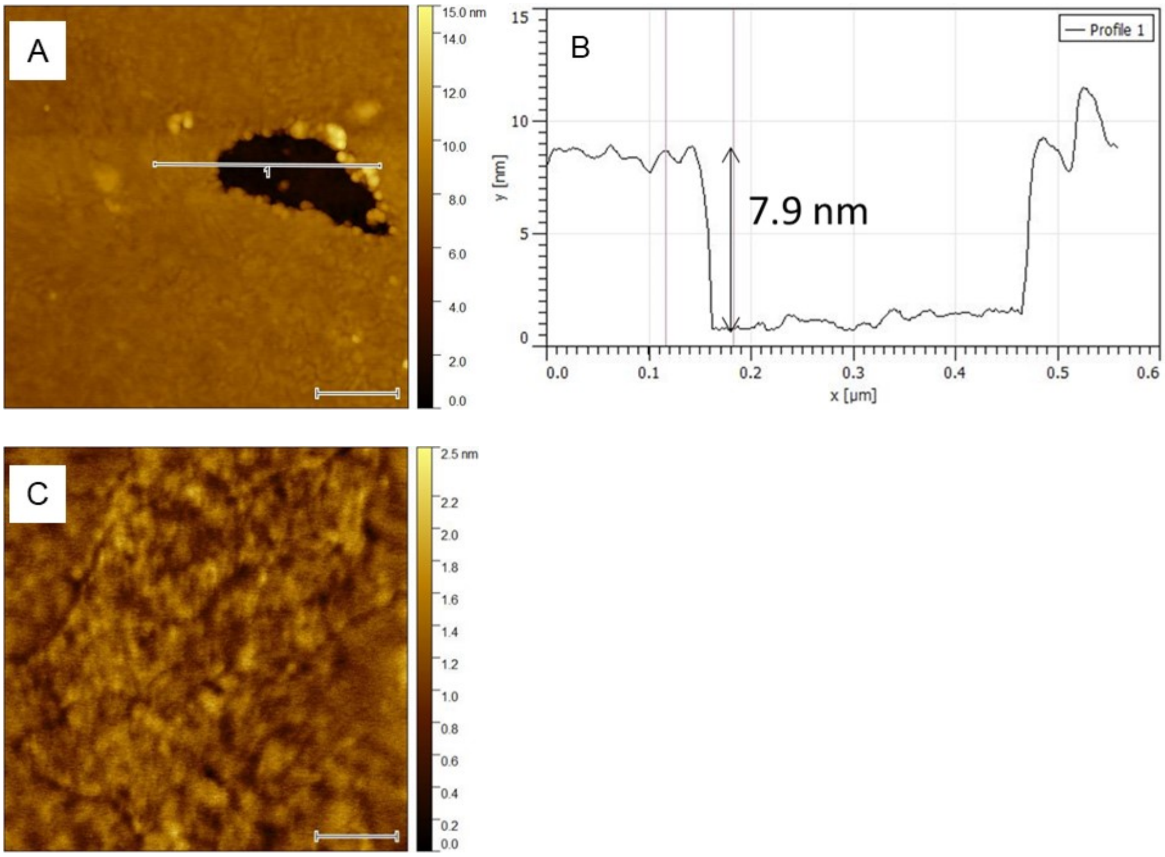

Figure S5. AFM of annealed 8 nm LT-TiO_2 on SiO_2/Si support after 72 h dissolution in 0.1 M $HClO_4$ pH 1. A) AFM height images 1 µm ×1 µm, black bar represents 200 nm, line 1 shows location of profile analysis B); C) AFM height image 0.5 µm × 0.5 µm, surface nanomorphology of TiO_2 outside the pinholes, black bar represents 100 nm.

Table S2. The electrochemical blocking properties of 8 nm ALD TiO_2 layers on FTO after 72 h exposition to 0.1 M $HClO_4$ pH = 1, and 0.1 M phosphate buffer pH = 8.

	**Dissolution solution**					
	**None**		**0.1 M $HClO_4$**		**0.1 M phosph. buffer pH 8**	
**sample**	EPA/ %	Defect type	EPA/ %	Defect type	EPA/ %	Defect type
LT-TiO_2 as dep.	7	B	47	A/B	29	B
LT-TiO_2 annealed	43	A/B	65	A	62	A/B
HT-TiO_2 as dep.	-	-	-	-	-	-
HT-TiO_2 annealed	17	A/B	24	B	27	B

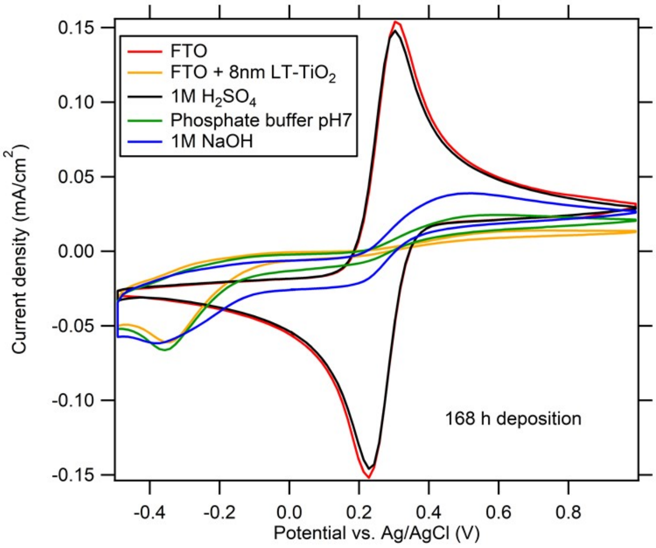

Figure S6. CVs of 0.5 mM $K_3[Fe(CN)_6]$ and 0.5 mM $K_4[Fe(CN)_6]$ in 0.5 M KCl on FTO electrodes covered with 8 nm LT-ALD TiO_2 as deposited, after dissolution in 1 M NaOH, 1 M H_2SO_4 and 0.1 M phosphate buffer pH 7 for 168 hours.

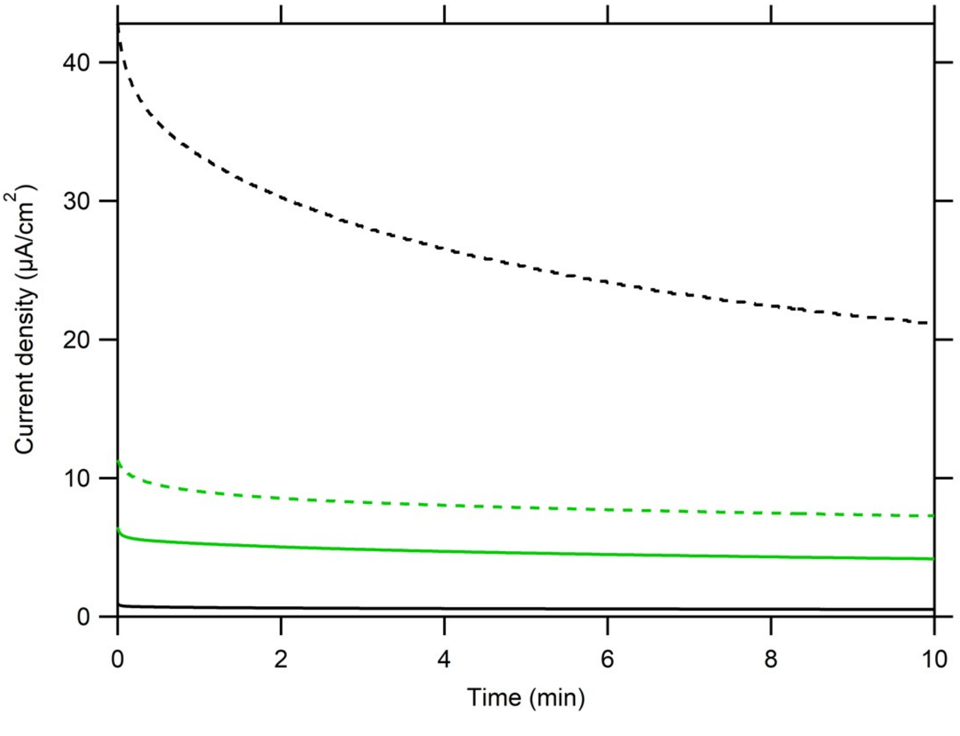

Figure S7. Time dependence of photocurrent of 8 nm TiO_2/FTO, electrolyte 0.1 M Na_2SO_4 (pH 10), applied potential 1 V vs. Ag/AgCl, irradiance 100 W/m^2, wavelength 369 nm. Black solid curve (LT-ALD TiO_2 as deposited), black dashed curve (LT - ALD TiO_2 annealed), green solid curve (HT-ALD TiO_2 as deposited), green dashed curve (HT-ALD TiO_2 annealed).

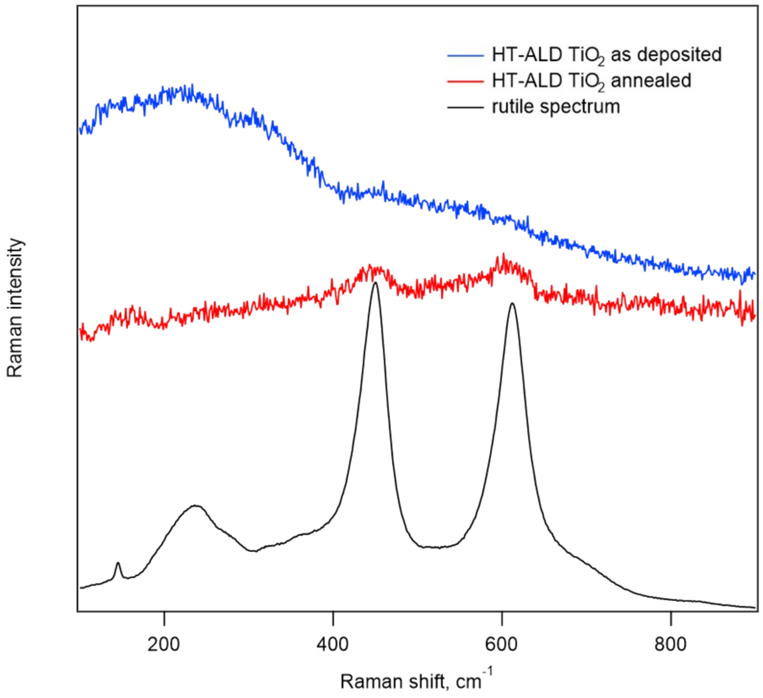

Figure S8. Raman spectra of 50 nm HT-ALD TiO_2/FTO as deposited (blue curve) and after annealing (red curve). The spectrum (black) of commercial rutile powder (Bayer) was added for comparison. The intensity of the last-mentioned is reduced by 0.05. The spectra are offset for clarity.

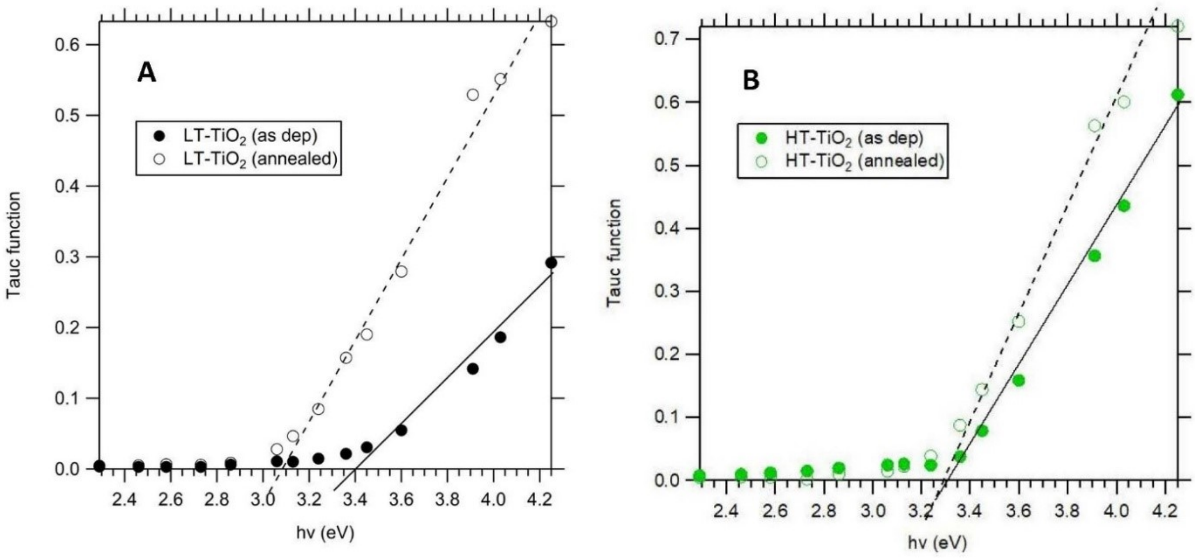

Figure S9. Electrochemical Tauc plot of LT-ALD TiO_2/FTO (A) and HT-ALD TiO_2/FTO (B). TiO_2 layer thickness was 8 nm. Black solid curve (LT-ALD TiO_2 as deposited), black dashed curve (LT - ALD TiO_2 annealed), green solid curve (HT-ALD TiO_2 as deposited), green dashed curve (HT-ALD TiO_2 annealed).
